# Cross-Scale Hypergraph Neural Networks with Inter–Intra Constraints for Mitosis Detection

**DOI:** 10.3390/s25144359

**Published:** 2025-07-12

**Authors:** Jincheng Li, Danyang Dong, Yihui Zhan, Guanren Zhu, Hengshuo Zhang, Xing Xie, Lingling Yang

**Affiliations:** 1 School of Artificial Intelligence and Computer Science, Nantong University, Nantong 226019, China; 2330110412@stmail.ntu.edu.cn (J.L.);; 2 School of Information Science and Technology, Nantong University, Nantong 226019, China; xiexing@ntu.edu.cn; 3 Xinglin College, Nantong University, Nantong 226019, China

**Keywords:** Thin-Prep cytologic test, cell detection, hypergraph neural network

## Abstract

Mitotic figures in tumor tissues are an important criterion for diagnosing malignant lesions, and physicians often search for the presence of mitosis in whole slide imaging (WSI). However, prolonged visual inspection by doctors may increase the likelihood of human error. With the advancement of deep learning, AI-based automatic cytopathological diagnosis has been increasingly applied in clinical settings. Nevertheless, existing diagnostic models often suffer from high computational costs and suboptimal detection accuracy. More importantly, when assessing cellular abnormalities, doctors frequently compare target cells with their surrounding cells—an aspect that current models fail to capture due to their lack of intercellular information modeling, leading to the loss of critical medical insights. To address these limitations, we conducted an in-depth analysis of existing models and propose an Inter–Intra Hypergraph Neural Network (II-HGNN). Our model introduces a block-based feature extraction mechanism to efficiently capture deep representations. Additionally, we leverage hypergraph convolutional networks to process both intracellular and intercellular information, leading to more precise diagnostic outcomes. We evaluate our model on publicly available datasets under varying imaging conditions, and experimental results demonstrate that our approach consistently outperforms baseline models in terms of accuracy.

## 1. Introduction

Breast cancer is the most commonly diagnosed cancer among women worldwide, accounting for 24.2% of all new cancer cases and 15% of cancer-related deaths in women [1,2]. Early detection of breast cancer significantly improves prevention and treatment outcomes. Currently, the most common and effective method for early screening is histopathological diagnosis using tissue sections. Among various histopathological features, mitotic activity is a key indicator of tumor aggressiveness [3,4]. Traditionally, pathologists manually examine hematoxylin and eosin (H&E)-stained slides to identify mitotic figures in gigapixel whole slide images (WSIs). This process is time-consuming and labor-intensive. Due to the global shortage of trained pathologists, manual diagnosis is often slow and costly.

**Figure 1:**
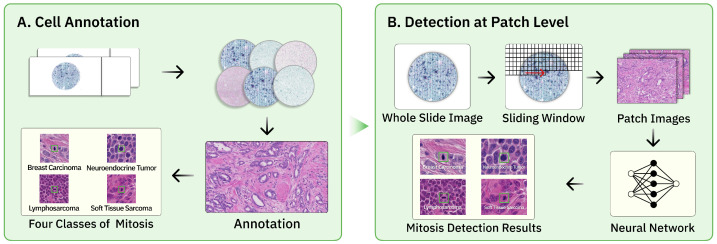
Data collection and training workflow. (**A**) After digitizing the hematoxylin and eosin (H&E)-stained specimens, pathologists annotate the types of mitosis in different tumors. (**B**) Using a sliding window, the WSI is cropped into patch-level images, which are then fed into a neural network for training to obtain the final results.

As deep learning technology improves by leaps [5], AI models for processing pathological images have made significant breakthroughs, offering new perspectives for mitosis detection [6]. As illustrated in Figure 1, the current pipeline for automated mitosis detection typically involves several key steps. First, whole slide images are acquired and preprocessed. Then, expert pathologists annotate the mitotic figures. Common mitotic lesions in different tumors include breast carcinoma, neuroendocrine tumor, lymphosarcoma, and soft tissue sarcoma. Subsequently, the WSIs are divided into hundreds of tiled image patches, and neural networks are employed to detect mitotic cells within these patches.

In recent years, numerous deep learning techniques have been developed for mitosis detection in different types of tumors. These methods can be categorized into two primary groups: the first group utilizes object detection networks that are effective on natural images, such as Faster R-CNN [7] and RetinaNet [8]. However, these approaches often struggle with performance and lack interpretability, making them unsuitable for clinical use. The second group includes enhanced detection techniques that incorporate specific knowledge about tumors. D. Cireşan et al. [9] proposed a simple convolutional neural network (CNN) for mitosis detection and won first place in the 2012 ICPR Mitosis Detection Challenge. He was among the first to apply deep learning techniques to the task of mitosis detection. H. Chen et al. [10] designed a deep cascade network that first performs coarse localization of candidate cells, followed by a fine-grained classification model that incorporates knowledge from cross domains. Alom et al. [11] introduced an integrated multi-block reference scheme along with a novel confidence analysis strategy to improve overall detection performance. Although these methods have achieved significant performance, they still face several critical challenges:

1. **Limited training data:** In pathological slides, mitotic cells are significantly fewer than normal cells, leading to a severe class imbalance between positive and negative samples in the dataset. Furthermore, since annotation requires expert pathologists, the amount of data available for training is extremely limited. In addition, the structure and morphology of cells vary greatly, and an imbalanced distribution among different types of mitotic figures is very common in datasets. As a result, trained detection models often suffer from high false negative or false positive rates. Moreover, with the increasing complexity and parameter size of current benchmark models, overfitting on mitosis detection tasks has become a frequent issue.
2. **Subtle features:** Mitosis is a complex biological process and pathologists typically rely on nuclear morphology to determine whether a cell is undergoing mitosis. In H&E-stained slides, mitotic nuclei appear as dark blue dots, which are often difficult to distinguish from the background and can easily be confused with apoptotic cells that also appear as dark blue dots. In addition, the morphological differences between various cell types in tissue sections are minimal, and mitotic cells often resemble normal cells in appearance. These factors make the accurate detection of mitotic figures extremely challenging.
3. **Neglecting cell relationships:** In pathological diagnosis, valuable information lies not only in individual cellular features but also in the relationships between surrounding cells. For cells with ambiguous or indistinct features, pathologists often rely on comparisons with neighboring cells to determine whether mitosis is occurring. However, most current models lack the ability to effectively model intercellular context, resulting in the loss of critical diagnostic information.

In addition, we surveyed the recent top-performing methods from the MIDOG2022 challenge. The winning solution, Stain-Robust Mitotic Figure Detection for the Mitosis Domain Generalization Challenge [12], formulated mitosis detection as a pixel-level segmentation task with stain-invariant masks, while the runner-up, Sk-Unet Model with Fourier Domain for Mitosis Detection [13], employed frequency-domain adaptation to address domain shift. Although both methods achieved strong accuracy, they rely on labor-intensive pixel annotations and do not explicitly model the clinical prior knowledge or intercellular context used by pathologists. These limitations highlight ongoing challenges in bridging algorithmic performance with practical diagnostic needs.

Nowadays, Graph Neural Networks (GNNs) are extensively utilized in multiple domains [14,15,16,17], leading to the development of various graph-based enhancements. One notable advancement is the hypergraph convolutional network (HGNN) introduced by Feng et al. [18], which is designed to manage complex data. Unlike conventional graph networks, HGNN enables a hyperedge to link several vertices, providing greater flexibility in data modeling. Building on this concept, we created an Inter–Intra Hypergraph Neural Network that achieves improved accuracy in mitosis detection. In conclusion, the key contributions of this paper are as follows:

1. Design a Block-Based Mixed Mechanism (BBMM), using parallel convolutional modules to efficiently extract deep information and enrich the gradient flow during training. In the feature fusion phase of the model, use a Bottom–Up mechanism to recover non-abstracted spatial details. These operations enable efficient feature analysis at the inter-level.
2. Thoroughly analyze the shortcomings of existing mitosis detection models and apply the HGNN concept to the cell detection domain, effectively modeling the relationships between individual cells and cell populations. A novel hyperedge convolutional construction is designed to capture the visual features of different cells. These operations facilitate efficient feature analysis at the intra-level.
3. Test the model on a public dataset with multiple tumor labels and originating from different staining imaging conditions, achieving favorable results. According to this research, this is the first time the HGNN concept has been applied in the mitosis detection domain.

## 2. Materials and Methods

### 2.1. Overview

The YOLO series [19,20,21,22,23,24,25,26,27,28,29,30,31] of single-stage object detection networks have been widely applied in natural image processing due to their accuracy and real-time performance. The best-performing model in this series is currently YOLO11 [32]. The architecture of the YOLO11 network can be divided into three components: the backbone network, which extracts features from images; the neck network, which fuses multi-scale features; and the head network, which generates local candidate boxes. Although the original YOLO11 algorithm demonstrates strong generalization capabilities, it has some limitations. It has difficulty detecting small objects and does not effectively model visual relationships at the group level [33]. These limitations render the original model inadequate for tasks that involve mitosis detection.

**Figure 2:**
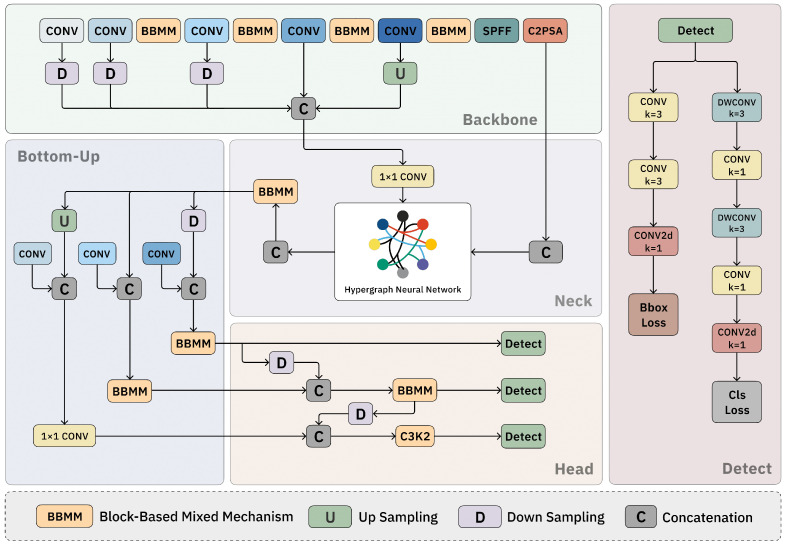
Architecture of the proposed mitosis detection network. The model enhances YOLO11 by introducing Block-Based Mixed Mechanism (BBMM) modules in the backbone, hypergraph neural network (HGNN) in the neck, and a Bottom–Up structure in the head. The network captures both individual cell features and intracell relationships to improve mitosis detection performance.

In order to address the limitations of the baseline model in mitosis detection, we propose an enhanced network architecture, which is depicted in Figure 2. Specifically, we introduce a Block-Based Mixed Mechanism (BBMM) within the backbone of the network. This mechanism employs a parallel convolution strategy which is designed to enrich the gradient flow during the training process. As a result, it significantly improves the feature representation at the inter-level, allowing for more robust and detailed extraction of cell features. In the neck of the network, we integrate hypergraph convolution networks. These networks are capable of effectively modeling the relationships between individual cells, thereby facilitating the fusion of heterogeneous visual features at the intra-level. This integration ensures that the network can capture both the individual characteristics of cells as well as their interactions with neighboring cells. Furthermore, in the head of the network, we incorporate a Bottom–Up strategy. This strategy enhances the network’s capability to process cross-hierarchical information, enabling it to better understand the context and structure of cell populations. By jointly leveraging the individual-level cellular attributes and the population-level contextual information, our proposed design produces a high-precision mitosis detection framework. This framework not only improves the representational capacity of the network, but also enhances its structural awareness, making it more effective in mitosis detection with high accuracy and reliability.

### 2.2. Block-Based Mixed Mechanism (BBMM)

**Figure 3:**
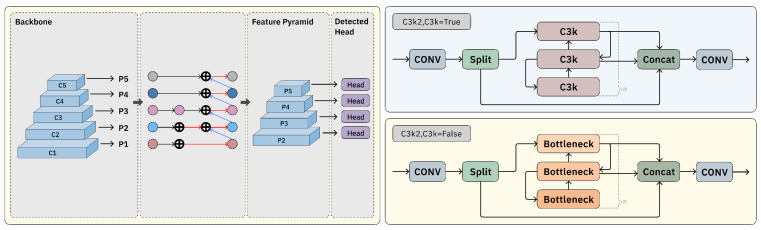
Architecture of the YOLO network modules. The upper part shows the structure of the standard C3K2 module; the lower part shows the Bottleneck module in the proposed Block-Based Mixed Mechanism (BBMM). The redesigned module enhances feature representation and multi-scale fusion capabilities.

As illustrated in Figure 3, the C3K2 module mainly relies on a single module for convolutional operations aimed at feature extraction. Although it supports both feature extraction and fusion, its capacity to capture a variety of information flows is somewhat restricted. Furthermore, when addressing multi-scale features, the C3K2 module primarily utilizes a basic fusion approach, which limits its effectiveness in integrating features across different levels. These shortcomings greatly hinder the model’s ability to handle intricate cellular visual information, making the original YOLO11 architecture insufficient for accurate cell detection tasks.

**Figure 4:**
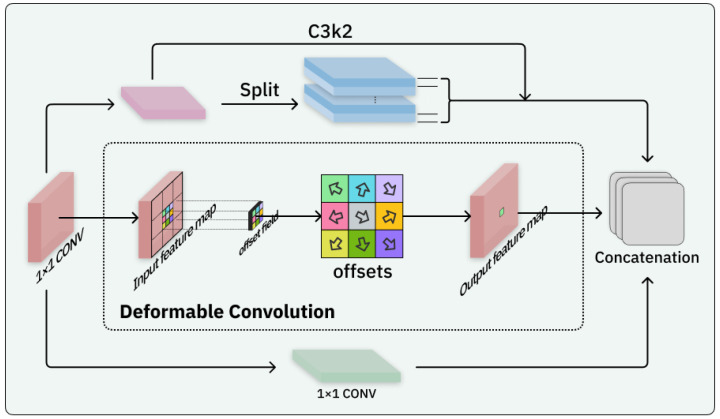
Architecture of the BBMM. The BBMM integrates the original C3K2 module with additional 1 × 1 convolution and deformable convolution branches to enhance both inter-channel feature interactions and adaptive spatial modeling.

Given the limited feature extraction capability of the C3K2 module, we attribute this limitation to its reliance on a single convolutional mechanism. To address this, we design the Block-Based Mixed Mechanism (BBMM). While retaining the original C3K2 module, BBMM incorporates two additional classic convolutional operations: 1 × 1 convolution and deformable convolution [34], as illustrated in Figure 4. This enhancement aims to enrich the representation of features by introducing both fine-grained channel transformations and adaptive spatial modeling.

The 1 × 1 convolution does not capture local spatial relationships within the input data but instead focuses on inter-channel interactions, allowing for channel-wise feature recalibration. To address the morphological variations of cells at different pathological stages, we propose using deformable convolution, which dynamically adjusts the receptive field to accommodate nonrigid deformations. By adopting a parallel strategy that integrates these three types of convolutions, we enhance the diversity and richness of the gradient flow during training, thus laying a stronger foundation for subsequent modeling in the neck module.

### 2.3. Hypergraph Neural Network

Our model refinement focuses primarily on the neck module, where we incorporate the concept of hypergraph convolution networks. Unlike traditional graph structures, where an edge connects only two vertices, a hypergraph allows a single hyperedge to connect multiple vertices simultaneously. The formal definition of a hypergraph is typically given as

(1) G=(V,E)

where V signifies the collection of vertices and E indicates the collection of hyperedges. In our approach, we decompose the grid-based visual features to create the vertex set V of a hypergraph. More specifically, the features obtained from five successive convolutional operations are combined through channel-wise concatenation to generate a hybrid feature representation $X_{m}$. For each feature map with dimensions $\left(C_{i},H_{i},W_{i}\right)$, where $C_{i}$, $H_{i}$ and $W_{i}$ are its channel, height, and width, respectively, the resulting dimension of the hybrid feature $X_{m}$ is

(2) $$C_{m}=\sum_{i=1}^{5}C_{i}.$$

where the upper limit 5 corresponds to the 5 successive convolutional outputs $F_{1}$–$F_{5}$ obtained from stages P3–P7 of YOLO11.

**Figure 5:**
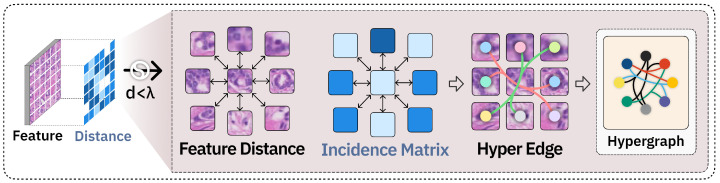
Architecture of the hypergraph neural network. Feature distances are computed between feature points to construct an incidence matrix and hyperedges, enabling the formation of a hypergraph that models intercellular relationships.

In a hypergraph, each feature point can be regarded as a vertex, which means that the total number of vertices corresponds to the total number of pixels on the feature map. With such a high number of vertices, a major challenge is to create an effective strategy for constructing hyperedges that can efficiently identify meaningful relationships between them. The construction method of the hypergraph can be visualized as shown in Figure 5.

We create the collection of hypergraph vertices denoted as V. To represent the neighborhood connections within the hypergraph module, we establish the set of hyperedges E using a distance threshold λ. For each feature point $x_{u}$, we identify all feature points that are within a distance of less than λ and connect them to form a hyperedge with $x_{v}$. A hyperedge *e* can be expressed as

(3) $$e=\{u|||x_{u}-x_{v}|{|}_{2}<\lambda ,u\in V\},$$

The Euclidean Norm ${∥\cdot ∥}_{2}$ encompasses all hyperedges that make up the hyperedge set *E*. The incidence matrix *H* for the hypergraph G=(V,E) is defined as follows:

(4) $$H_{ve}=\left\{\begin{array}{cc} 1, & \mathrm{if}\;v\in e\\ 0, & \mathrm{if}\;v\notin e \end{array}\right.$$

In hypergraph convolutional networks, increasing the number of layers can lead to a gradual loss of information during propagation. To mitigate this issue, we incorporate residual connections that allow the model to transmit input information directly to the output, thereby reducing the risk of information loss. To facilitate the propagation of high-order information within the hypergraph structure, we employ spatial hypergraph convolution along with residual connections. The computation process is outlined as follows:

(5) $$\left\{\begin{array}{c} X_{e}=\frac{1}{|N_{v}\left(e\right)|}\sum_{v\in N_{v}\left(e\right)}X_{v}\Theta_{e},\\ X_{v}^{\prime }=X_{v}+\frac{1}{|N_{e}\left(v\right)|}\sum_{v\in N_{e}\left(v\right)}X_{e} \end{array}\right.$$

Let $N_{v}\left(e\right)$ denote the neighborhood of vertices associated with the hyperedge *e*, and $N_{e}\left(v\right)$ denote the neighborhood of hyperedges linked to the vertex *v*. Additionally, $\Theta_{e}$ is a parameter that can be trained. With a feature matrix *X* for the vertices and an adjacency matrix *H* for the hypergraph, and assuming $D_{v}$ and $D_{e}$ are the degree matrices for the vertices and hyperedges, respectively, the hypergraph convolution can be formulated as follows:

(6) $$HyperConv\left(X,H\right)=X+D_{v}^{-1}HD_{e}^{-1}H^{T}X\Theta$$

The expression $D_{v}^{-1}HD_{e}^{-1}$ computes the normalized adjacency matrix, while H is the adjacency matrix of the graph. $D_{v}^{-1}HD_{e}^{-1}H^{T}X$ aggregates vertex features via hyperedges to identify higher-order connections among vertices. The matrix Θ is a learnable parameter that transforms the aggregated features, improving the expressiveness of the model. Additionally, a residual connection of *X* is included to maintain the original information about the feature and avoid any loss of information.

In summary, the hypergraph neural network addresses the shortcomings of conventional grid structures, improving the depth and detail of feature representations. This approach effectively merges individual-level inter-correlation features of cells with population-level intra-discriminative features, allowing for a successful integration of diverse features.

### 2.4. Bottom–Up

After defining the vertex set V and the hyperedge set E, we proceed to build the hypergraph convolution network. The output of this network encompasses higher-order, cross-level information. To better align with the head and improve detection capabilities, we implement a Bottom–Up mechanism [35] between the neck and head. As features move through deeper layers, spatial details tend to diminish due to the more abstract nature of the feature representations. As illustrated in Figure 2, the information processed by the hypergraph is integrated with the last three layers of the backbone’s output. This integration allows for the transfer of high-resolution details from the shallower layers to the deeper layers, thereby maintaining essential structural information and offsetting the loss of spatial details in the baseline.

## 3. Experiments and Results

### 3.1. Datasets

The evaluation dataset used in our experiments is the MIDOG2022 dataset [36] provided by the MICCAI Challenge. This dataset is specifically designed for detecting mitosis in different types of tumor cells. The MIDOG dataset contains 150 whole slide images (WSIs) annotated with various types of mitosis. These WSIs were captured using three different imaging conditions with scanners, each providing 50 WSIs. This necessitates higher robustness in the detection models due to the variability in imaging conditions. To facilitate network input, we performed preprocessing operations on all the images, dividing each complete WSI into patches of 640 × 640 pixels and then reconstructing the lesion annotations for each patch.

In total, the MIDOG2022 dataset includes 9501 annotated mitotic figures and 11,051 hard negative samples, ensuring a rich yet challenging training environment. Each WSI belongs to one of several tumor types, including breast carcinoma, lymphoma, lung carcinoma, melanoma, mast cell tumor, and neuroendocrine tumor, drawn from both human and canine subjects. These categories are approximately balanced within the mitotic class, but the overall dataset remains highly imbalanced, with mitotic figures constituting less than 10% of all annotated cells. To prevent data leakage, the dataset is split at the slide level into training (70%, 105 WSIs), validation (15%, 22 WSIs), and testing (15%, 23 WSIs) sets. After preprocessing, this resulted in approximately 8000 mitosis-positive patches and over 100,000 background patches. The substantial class imbalance and diversity in both tissue origin and imaging conditions make MIDOG2022 an ideal benchmark for evaluating model robustness and generalization.

It is worth noting that the specific cancer types corresponding to each WSI have not been disclosed by the dataset organizers, in order to avoid introducing potential bias. As a result, no tumor-specific stratification was applied during training or evaluation. Nevertheless, the dataset includes mitoses sampled from a variety of tumor tissues and scanning conditions, ensuring broad diversity in visual patterns and clinical relevance.

### 3.2. Implementation Details

The implementation of our method is based on the PyTorch framework (version = 1.8.0) and runs on an NVIDIA RTX 3090 GPU (Leadtek Research Inc., Shanghai, China). Our model is trained using the YOLO11n pre-trained model with the Adam optimizer [37]. The comparative experiments are conducted using MMDetection [38] and evaluated with COCO-style [39] metrics for quantitative analysis. These metrics include P (Precision), R (Recall), and mAP (Mean Average Precision). The specific calculation formulas for these metrics are as follows:

(7) $$\mathrm{Precision}=\frac{\mathrm{TP}}{\mathrm{TP}+\mathrm{FP}}$$

(8) $$\mathrm{Recall}=\frac{\mathrm{TP}}{\mathrm{TP}+\mathrm{FN}}$$

(9) $$\mathrm{AP}=\int_{0}^{1}P\left(R\right)\;dR$$

(10) $$\mathrm{mAP}=\frac{1}{C}\sum_{i=1}^{C}{\mathrm{AP}}_{i}$$

(11) $$\mathrm{ACC}=\frac{\mathrm{TP}+\mathrm{TN}}{\mathrm{TP}+\mathrm{FP}+\mathrm{FN}+\mathrm{TN}}$$

(12) $$F_{1}\text{-}\mathrm{score}=2\cdot \frac{precision\cdot recall}{precision+recall}$$

True positive (TP) refers to the count of instances that the model correctly classifies as positive. False positive (FP) is the number of negative instances that are incorrectly identified as positive by the model. False negative (FN) represents the instances where the model incorrectly classifies a positive instance as negative. True negative (TN), conversely, denotes the number of negative instances that are correctly identified as negative. Precision is derived by dividing TP by the total of TP and FP, offering a measure of the model’s accuracy in predicting positive outcomes. Recall is the ratio of TP to the sum of TP and FN, indicating the model’s capability in detecting positive instances [40]. Average Precision (AP) is the area under the Precision–Recall (P-R) curve [41], which can be computed by integrating the curve. The constant C indicates the number of categories. In multi-class classification, AP is computed for each class individually, and the average of these AP values yields the mean Average Precision (mAP), which accounts for the specific P-R curve of each class. Overall Accuracy (ACC) measures the proportion of all correctly classified samples—both positive (TP) and negative (TN)—to the total number of samples, providing a global indicator of recognition performance. Finally, the F_1_-score is the harmonic mean of Precision and Recall, balancing the trade-off between FP and FN to summarize a model’s effectiveness when both error types are of comparable concern.

### 3.3. Comparative Analysis

To ensure a comprehensive evaluation, we compare our method with several representative CNN-based detectors, including Faster R-CNN, RetinaNet, YOLOX, YOLO11, Sparse R-CNN, Cascade R-CNN, and RT-DETR. These models span classical two-stage frameworks, efficient single-stage detectors, and transformer-based architectures. Faster R-CNN and Cascade R-CNN are well-established two-stage models with strong localization performance. RetinaNet and YOLOX are popular one-stage detectors, with RetinaNet addressing class imbalance via Focal Loss. RT-DETR leverages transformer-based global attention for enhanced accuracy, while YOLO11 represents the latest YOLO variant balancing speed and precision. Collectively, these baselines form a diverse and rigorous benchmark for validating our approach. We strictly adhere to the rules for quantitative analysis, and the results of our tests on the same GPU are presented in Table 1. Here, *mAP50* denotes the mean Average Precision computed at an IoU threshold of 0.50, *Paramets* (*M*) indicates the number of trainable parameters expressed in millions, and *GLOPs* (*G*) refers to the computational cost in giga floating-point operations for a single forward pass. Please note that the YOLO11 version used here is YOLOv11-l.

It can be observed that on the MIDOG2022 dataset, our proposed detection model consistently outperforms other object detection models. In particular, our model achieves a 6.4% improvement in mAP50 compared to the existing best model, which is the result of more accurate localization and classification by our model. Furthermore, our model improves in Precision and Recall by 2.8% and 5.2%, respectively. It is worth noting that all methods are trained solely on the official MIDOG2022 training set, which contains 150 whole slide images (WSIs) with about 9000 annotated mitotic figures—considerably smaller than pathological benchmarks such as CAMELYON17 [46], which comprises 1000 WSIs with tumor-region annotations, or DeepLesion [47], which includes more than 32,000 CT lesions. Hence, the superior results indicate that our model can maintain high detection accuracy even with limited training data, making it particularly suitable for fields such as mitosis detection, where data acquisition is challenging.

To further visualize the classification effectiveness, we compare the confusion matrices of the baseline model and our proposed approach. These matrices reveal how each model distinguishes between mitotic figures, hard negatives, and background, providing complementary insight to the numerical metrics reported earlier.

**Figure 6:**
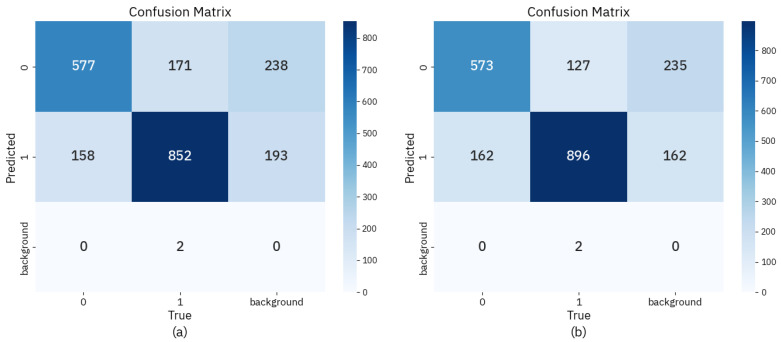
Confusion matrix: (**a**) baseline; (**b**) ours.

The confusion matrices in Figure 6 offer an intuitive comparison of the baseline model and our proposed approach. In contrast to the baseline, our model evidently demonstrates improved classification accuracy. Specifically, the number of correctly identified mitotic figures increases by 44 instances, while false positives related to background and hard negatives are significantly reduced. This improvement indicates that our model not only enhances mitosis detection sensitivity but also suppresses over-detection of non-mitotic structures. The reduction in misclassification between morphologically similar classes further suggests better inter-class separability. These results align with the Precision and Recall gains reported in Table 1, underscoring our model’s robustness in handling visually ambiguous samples.

### 3.4. Ablation Study

To thoroughly assess the effectiveness of each component in our proposed network architecture, ablation studies were conducted using the MIDOG2022 dataset. As illustrated in Table 2, our method consistently demonstrates substantial improvements across various evaluation metrics when compared to the baseline YOLO11-l model.

Specifically, after the introduction of the Block-Based Mixed Mechanism (BBMM) module, the model exhibits notable enhancements across all metrics. This clearly indicates that the parallel multi-convolutional structure within the BBMM module significantly boosts the model’s ability to capture and model complex cellular structures, thereby enriching the feature representation at the inter-level. Subsequently, upon incorporating the Hypergraph Convolutional Neural Network (HGNN) module, the model achieves remarkable improvements in both Average Precision (AP) and Average Recall (AR). This finding underscores the critical importance of modeling the intercellular structural relationships for the accurate recognition of high-heterogeneity lesions. By effectively capturing the interactions between cells, the HGNN module further enhances the model’s intra-level feature fusion capabilities. Furthermore, when both the BBMM and HGNN modules are integrated into the network, the system’s overall performance reaches its peak. The mean Average Precision (mAP) increases from 87.5 to 93.6, highlighting the synergistic benefits of combining inter-level and intra-level modeling. This substantial improvement demonstrates that the joint optimization of individual cell features and population-level contextual information significantly boosts the model’s detection accuracy and robustness.

**Figure 7:**
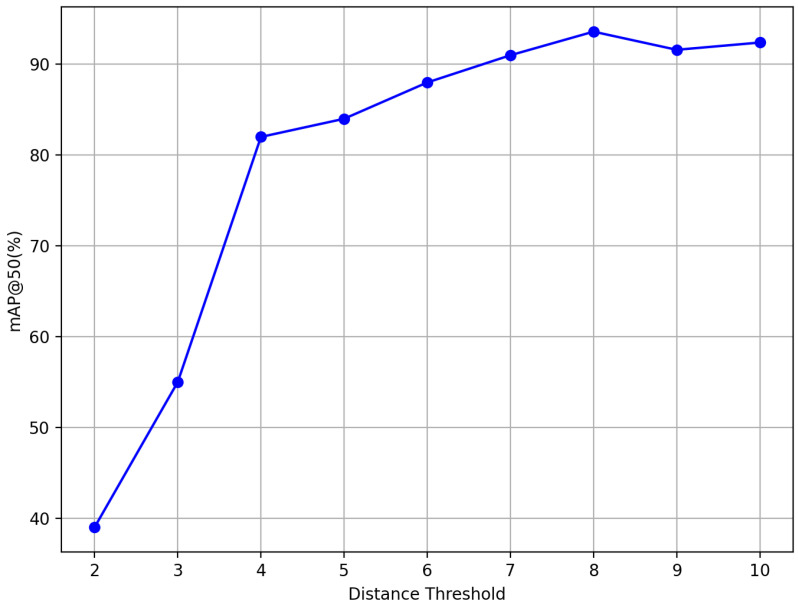
Ablation study on the distance threshold.

It is worth noting that adjusting the detection threshold is crucial for performance optimization. In neural networks, performance related to information propagation typically follows a convex curve, indicating the existence of an optimal threshold. Empirical analysis can help determine a reasonable range for the threshold, while ablation studies can assist in identifying the precise value. The specific experimental results are shown in Figure 7.

Based on the experimental results, the performance of the proposed network follows a convex curve. As the threshold increases, the detection performance increases rapidly and remains relatively stable with only minor fluctuations in the threshold range of 7 to 10. We attribute this increase to the fact that lower thresholds result in reduced hypergraph connectivity, which does not fully take advantage of the high-order relationships among features. Conversely, higher thresholds connect more hypergraphs, leading to excessive information sharing and over-smoothing of features. This is evident in the performance drop observed when the threshold is set to 9, as shown in the figure. Therefore, our detection network is constructed using a distance threshold of 8.

### 3.5. Visualization Map

**Figure 8:**
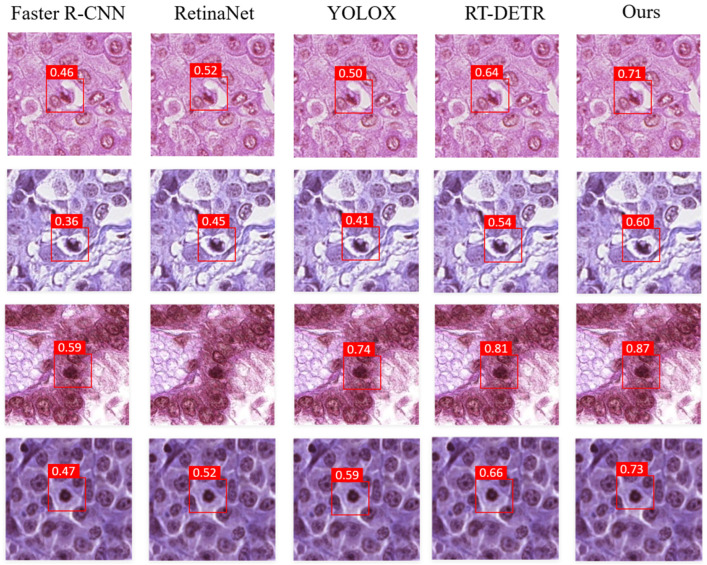
Comparative experiment visualization map.

To intuitively demonstrate the accuracy of our model, we selected several representative comparison samples, as shown in Figure 8. Please note that due to the large image sizes, we cropped local regions for better visualization. The number inside each bounding box denotes the confidence score (0–1) output by the corresponding detector for the “mitotic figure” class; the larger the value, the more certain the model is about that prediction. Specifically, we have drawn the following two conclusions: (1) First, among the existing object detection models, the RT-DETR model generally outperforms the others, demonstrating the powerful capabilities of Transformer-based models. (2) Second, our model achieves better detection results than RT-DETR. When dealing with the same mitotic cells, it can display higher confidence levels. Our method successfully identifies subtle lesion patterns that are often missed or misclassified by general-purpose object detection models. Combined with the comparative experiments, this highlights the superior classification accuracy of our model.

(13) $$\mathrm{Confidence}=\mathrm{Pr}\left(\mathrm{Object}\right)\times {\mathrm{IOU}}_{\mathrm{truth}}^{\mathrm{pred}}$$

where Pr(Object) reflects the classification accuracy for the “mitotic figure” class, and ${\mathrm{IOU}}_{\mathrm{truth}}^{\mathrm{pred}}$ represents the localization accuracy between the predicted box and the ground truth.

## 4. Conclusions

This study provides a systematic analysis of the key challenges in mitosis detection and the limitations of existing algorithms, and innovatively proposes an intelligent detection algorithm based on an Inter–Intra dual-stream architecture. At the feature extraction level, a parallel convolution module is designed to efficiently encode block-level features, and a multi-path gradient propagation mechanism is employed to enhance the representation ability of deep information. In the feature fusion stage, a Bottom–Up reverse refinement strategy is introduced to effectively retain spatial detail information from the original image, constructing a multi-scale feature parsing framework at the inter-level. To address the inadequate modeling of complex intercellular associations, this study introduces, for the first time, the hypergraph neural network (HGNN) into the cell detection domain. Through a learnable hyperedge construction mechanism, HGNN captures the heterogeneous relationships between individual cells and cell groups, forming a higher-order semantic reasoning network at the intra-level. As the first study to apply hypergraph learning to mitosis detection, the experiments show that the proposed method achieves excellent detection performance on public datasets, providing a new technological path for high-throughput mitosis detection.

The limitations of the proposed method and potential remedies are as follows. Imaging and staining conditions vary between different institutions, and a model trained in one dataset may not perform well on another dataset. To address this issue, future research will explore the adoption of an unsupervised staining normalization approach to integrate into the model. In addition, we will investigate the applicability of the proposed detection model to the analysis of pathological images for the diagnosis of other diseases.

## Figures and Tables

**Table 1:** The results of mitosis detection for different models.

Model	Precision (%)	Recall (%)	mAP50 (%)	ACC (%)	F1-Score	Paramets (M)	GLOPs (G)
Faster R-CNN [7]	74.5	78.2	82.0	74.6	0.763	41.3	190.1
RetinaNet [8]	68.2	70.5	72.3	68.2	0.693	37.9	193.8
Sparse R-CNN [42]	71.4	77.1	80.9	71.4	0.741	107.3	150.7
Cascade R-CNN [43]	79.3	85.1	88.4	79.3	0.821	77.3	278.4
YOLOX [44]	73.7	78.2	86.1	73.7	0.759	47.1	115.6
YOLO11 [32]	77.4	81.6	87.6	77.4	0.794	25.3	86.9
RT-DETR [45]	80.3	84.5	87.2	80.3	0.823	42	136
Ours	83.1	89.7	93.6	83.6	0.863	56.3	211

**Table 2:** Result of ablation experiments.

Model	BBMM	HGNN	Precision (%)	Recall (%)	mAP50 (%)
Baseline [32]			77.4	81.6	87.5
Baseline + BBMM	✓		80.4	86.9	88.2
Baseline + HGNN		✓	79.1	87.3	90.1
Ours	✓	✓	83.1	89.7	93.6

Note: ‘✓’ indicates that a corresponding improvement has been made.

## Data Availability

Data will be made available on request.
